# Metabolic Alterations in Male-Sterile Potato as Compared to Male-Fertile

**DOI:** 10.3390/metabo9020024

**Published:** 2019-02-01

**Authors:** Maria Shishova, Roman Puzanskiy, Olga Gavrilova, Shamuhommed Kurbanniazov, Kirill Demchenko, Vladislav Yemelyanov, Galina Pendinen, Alexey Shavarda, Tatjana Gavrilenko

**Affiliations:** 1 St. Petersburg State University (SPbGU), St. Petersburg 199034, Russia; mshishova@mail.ru (M.S.); puzansky@yandex.ru (R.P.); shamukurbanniyazov@rambler.ru (S.K.); bootika@mail.ru (V.Y.); stachyopsis@gmail.com (A.S.); 2 Federal Research Center, Vavilov All-Russian Institute of Plant Genetic Resources (VIR), St. Petersburg 190000, Russia; pendinen@mail.ru; 3 Komarov Botanical Institute, Russian Academy of Sciences (BIN), St. Petersburg 193022, Russia; olgaangav@gmail.com (O.G.); Demchenko@binran.ru (K.D.);

**Keywords:** *Solanum tuberosum*, potato, male sterility, untargeted metabolomics, metabolite profiles

## Abstract

The common potato, *Solanum tuberosum* L., is the fourth most important agricultural crop worldwide. Until recently, vegetative propagation by tubers has been the main method of potato cultivation. A shift of interest to sexual potato reproduction by true botanical seeds is due to the appearance of a new hybrid seed breeding strategy whose successful application for many crop species has been supported by male sterility. This investigation was focused on the study of differences in the metabolite profiles of anthers at the mature pollen stage from male-fertile and male-sterile genotypes of *S. tuberosum*. Application of gas chromatography coupled with a mass spectrometry method allowed detection of metabolic profiles for 192 compounds. Further data analysis with several libraries fully identified 75 metabolites; a similar amount was defined up to the classes. Metabolic profiles in the anthers of fertile genotypes were significantly distinguished from male-sterile ones by the accumulation of carbohydrates, while the anthers of sterile genotypes contained a higher amount of amino acids. In comparison with male-fertile plants, male-sterile genotypes had undeveloped pollen grain characters; i.e., smaller grain size, a thicker exine, “permanent tetrads” that failed to disintegrate into microspores, and the absence of pollen apertures that might be due to a disorder in the metabolism of carbohydrates and fatty acids.

## 1. Introduction

Many plants can reproduce only sexually. This type of reproduction provides a source of genetic variation and generates material for adaptation to a rapidly changing environment. At the same time, there are plant species that can also reproduce asexually through vegetative propagation. Asexual reproduction produces individuals genetically identical to the parent offspring, a feature which is beneficial under stable conditions.

The common potato, *Solanum tuberosum*, is the fourth most important agricultural crop worldwide. It is able to reproduce both sexually and asexually, though until recently, vegetative propagation by tubers has been the main method of cultivation.

A shift of interest from vegetative propagation to sexual reproduction by botanical seed (true potato seed – TPS) is due to the appearance of a new hybrid seed breeding strategy which includes: development of diploid inbred lines propagated by true seeds, their involvement in crosses, and the production of heterotic F_1_ hybrid cultivars that are genetically identical [1,2]. The expected advantages of hybrid seed technology in the potato are an increase in the efficiency of the selection and stacking of desirable alleles at the diploid level, and also a decrease in the risk of plant contamination, because most potato pathogens are not transmitted with pollen and TPS [1,2].

Successful application of F_1_ hybrid seed breeding for many crop species was supported by male sterility; i.e., cytoplasmic male sterility (CMS) and/or nuclear (genic) male sterility (NMS), which allow for avoidance of emasculations. Currently mass production of potato hybrid true seeds is mainly done by hand pollination [3]. At the same time, several sterile cytoplasm types are known for the potato that exhibit a different phenotypic appearance of male sterility traits. They are associated with undeveloped male reproductive organs (called anthers) (Т/beta cytoplasm), functional pollen sterility (D type) and tetrad sterility (W/gamma cytoplasm type) [4]. The finding of male-fertile T/beta- and D-cytoplasm type genotypes [5] could indicate the presence of the hidden male fertility *Restorer* gene(s). However, there is no information about CMS-*Rf* genetic systems in the potato [6].

One of the major potato cytoplasm types, W/gamma [7], has always been associated with complete male sterility which is expressed as “tetrad sterility” [8], when tetrads fail to disintegrate in microspores (permanent tetrads). This specific type of male sterility was introduced into potato cultivars from the Mexican species *S. stoloniferum* together with the nuclear *Ry_sto_* gene conferring resistance to the most damaging of the potato virus (PVY) [9]. Thus, the cause of “tetrad sterility” in the potato results from nuclear-cytoplasmic conflict [10,11] between the nuclear loci of the cultivated potato and W/gamma cytoplasm from a wild polymorphic species *S. stoloniferum*. Another cytoplasm type W/α(D), also found in *S. stoloniferum*, is not related to male sterility, as it has been detected in male-fertile genotypes [12].

Similar “permanent tetrads” phenotypes have been found in male-sterile mutants of the *Arabidopsis thaliana* [13,14], tomato (*Solanum lycopersicum*) [15], rapeseed (*Brassica napus*) [16], *Allium* species [17,18], and soybean (*Glycine max*) [19]. Some of these male-sterile mutants are considered candidates for hybrid seed breeding [15]. It has been shown that these mutations result in the failure of microspore separation and have predominantly affected genes regulating metabolic processes during anther development.

There is not much known about the genetic basis of “tetrad sterility” in the potato as well as the metabolomic changes that lead to this specific type of male sterility. In a number of investigations, carbohydrates were estimated to play an important role in anther and pollen development. Changes in sucrose and starch concentration in anthers positively correlated with modulation of the activity of several enzymes of carbohydrate metabolism as well as sucrose transporters [20,21,22,23]. Interestingly, tissue-specific antisense repression of extracellular invertase in tobacco plants resulted in an inhibition of pollen development at early stages, which triggered male sterility [21]. This approach was considered a powerful tool toward the regulation of male sterility.

In the present investigation, the GS-MS method was employed to reveal metabolic alterations in eight tetraploid potato accessions, *S. tuberosum*, distinguished by male fertility/sterility characteristics. Male-sterile potato genotypes were characterized by the formation of “permanent tetrads”. Such a phenomenon is also known in other species assumed to be dependent on callose (β-1,3-glucan) deposition during meiosis and the completion of its degradation is crucial for the formation of functional microspores [14,24]. Another important pollen cell wall component, sporopollenin, is a highly resistant biopolymer, which consists of phenolic compounds and long-chain aliphatic acids [25,26,27]. The dynamics of possible metabolic precursors of these polymers were tested as well as morphological cell wall (CW) rearrangements in anthers at the mature pollen stage in fertile and male sterile genotypes.

## 2. Materials and Methods

### 2.1. Plant Material

Plant material included eight tetraploid potato accessions, *Solanum tuberosum*, from the VIR (Federal Research Center, Vavilov All-Russian Institute of Plant Genetic Resources) collection. Four cultivars (cv.) and four breeding lines were selected for this study based on male fertility/sterility characters, cytoplasm type and their pedigree records (Table 1). Four genotypes (cv. Evraziya, cv. Gusar, cv. Sudarynja, breeding line 1604/16) do not produce fertile pollen; they are characterized by male tetrad sterility and sterile cytoplasm type W/gamma. The other four genotypes (cv. Lomonosovskij, breeding lines: 1101/10, 2103/7, 211/9) are male-fertile with cytoplasm type W/alpha(D) [28,29].

All these cultivars and breeding lines have different *S. stoloniferum* hybrids in their pedigrees; they have been selected earlier for their resistance to pathogens and good agronomic characters [30]. Cultivar Evraziya and breeding lines 1604/16, 1101/10, 211/9 have a common maternal or paternal parental lines in their pedigrees (Table 1). Cultivars Gusar, Sudarynja, Lomonosovskij each have an independent origin.

### 2.2. Sample Preparation

Each genotype was sampled in three biological replicates (individual plants). Flower buds 10 mm long (measured from the pedicel insertion point to the tip of the bud) with colored petals and yellow anthers were collected from the first inflorescence of individual plants for a metabolic analysis. These flower buds (two days before opening) contain completely developed and mature pollen. Anthers were isolated from the flower buds of all genotypes at the same time and fixed by liquid nitrogen immediately after they were separated from the floral buds and weighed. Parallel fixation of flower buds at different stages of development (I and II meiotic division, mature pollen grains) was carried out from the same plants for cytological observation and scoring pollen fertility.

Anthers frozen in liquid nitrogen were disrupted in a bead mill (Tissue Lyser LT, beats per second, three times for 2 min). Extraction was provided with 80% methanol. After extraction, the samples were purified from tissue debris by centrifugation for 10 min at 15,000 *g*. The resulting extract was treated with a vacuum evaporator. The dry sediment was then dissolved in pyridine containing the internal standard nC23 (tricosan), then a silylating agent was added in the proportion BSTFA (N,O-Bis(trimethylsilyl)trifluoroacetamide): TMCS (Trimethylchlorosilane) as 99:1 and the samples were derivatized by incubating them at 90 °C for 20 min.

### 2.3. Gas Chromatography Coupled with Mass Spectrometry (Gc-Ms)

For the GC-MS analysis, the Agilent 5860 gas chromatograph was used under the control of AgilentChemStation software E.02.02.1431. Sampling was performed using the autosampler Agilent 7893 in the “splitless” mode, 1 µL volume was injected. Separation was performed on a J&W HP-5MS capillary column (30 m in length, 0.25 mm in diameter, fixed phase film thickness (5% biphenyl, 95% dimethyl polyoxane) 0.1 μm). The carrier gas was helium, with a constant flow of 1.3 mL/min, and the evaporator temperature was 250 °C. Column thermostat temperature: base temperature was 70 °C increased at a speed of 4 °/min to 320 °C and then sustained for 10 min. The chromatogram was recorded by a Agilent 5975C mass-selective detector with *m*/*z* range 50–850. The source temperature was 230 °C.

### 2.4. Metabolite Identification and Quantification

The results of the chromatographic analysis were processed with the PARADISe program (Department of Food Science, Faculty of Science, University of Copenhagen, Copenhagen, Denmark, http://www.models.life.ku.dk/paradise) [31] in association with an NIST MS Search (National Institute of Standards and Technology (NIST), Gaithersburg, MD, USA). In addition, we used the AMDIS (Automated Mass Spectral Deconvolution and Identification System, NIST, Gaithersburg, MD, USA). The following mass-spectrometer libraries were used: NIST2010, library of the Research Park ‘Centre for Molecular and Cell Technologies’ (St. Petersburg State University, St. Petersburg, Russia), the Golm Metabolome Database (GMD), and MoNA (Massbank of North America). The retention index (RI) was determined by calibration with standard alkanes. Metabolites were quantified with PARADISe accordingly [31].

### 2.5. Statistical Analysis of Metabolomic Data

The analysis was provided within the R language environment 3.4.2 [32]. For quantitative interpretation, the data were normalized by the internal standard (nC23) and calculated per mass. Outlying values were excluded on the basis of Dixon’s test. When metabolite was not detected, or concentration excluded as an outlier but presented in other replicated samples, it was postulated as a technical error and missing values were imputed. Missing data imputation was performed by KNN (k-nearest neighbors) with the “impute” R package [33]. The data were standardized and log-transformed. A heatmap was made by the package ComplexHeatmap [34]. PCA (Principal Component Analysis) and its nonlinear neural network modification were realized with pcaMethods [35]. Kernel PCA (kPCA) [36] was performed with kernlab package [37]. LLE (Locally Linear Embedding) was performed with RDRToolbox [38]. Random Forest (RF) was hold by randomForest [39]. (O)PLS-DA was made with ropls [40]. Metabolites were mapped by significant (*p* < 0.05) and strong correlation coefficients (|r| > 0.7) of their arbitrary content within the software environment of Cytoscape [41], using the “organic layout”. Analysis of variance using distance matrices with a permutation test (PERMANOVA) [42] was performed with package vegan [43] using a Euclidian space of the first two PCs and 999 permutations.

### 2.6. Assessment of the Frequency of Abnormalities in the First and the Second Meiotic Divisions

Flower buds of male-sterile and male-fertile potato genotypes were fixed in ethanol-acetic acid solution (3:1 *v*/*v*) in room temperature over 24 h. The fixated material was stored in 70% ethanol until analysis. Appropriate-sized anthers were stained by 2% acetoorcein and the squashed slides were prepared. The frequencies (%) of irregularities were studied at different phases of meiosis by light microscopy.

### 2.7. Pollen Viability Assay

Pollen was stained in an Alexander solution and observed by light microscopy [44].

### 2.8. Aniline Blue Staining

For callose staining, samples were fixed in ethanol/acetic acid (3:1, *v*/*v*) and stained with 0.01% (w/v) aniline blue in 0.077 M phosphate buffer, pH 8.5, at room temperature for 10 min [45]. Callose deposition was visualized with laser microscopy (405 nm excitation, [14]). Fluorescence spectrums of stained and non-stained anthers were detected with a Carl Zeiss Laser Spectral Imaging Microscope LSM 780 (facility of Resource Center of Botanical Institute RAS). Exine possesses autofluorescence, which made it possible to study CW morphology with a fluorescent microscope, including CLSM, without additional staining.

### 2.9. Measurement and Morphological Observation of Pollen Grains

For exine thickness measurement (in µm) and calculation of the number of apertures, pollen grains were acetolysed [46], mounted in glycerine jelly, and sealed with paraffin. Exine thickness was measured on pollen grains lying in equatorial view and away from apertures. Pollen grains have been studied under light microscopy (LM) and confocal laser scanning microscopy (CLSM) Zeiss LSM 780 (Carl Zeiss AG, Oberkochen, Germany) [47].

## 3. Results

### 3.1. Metabolome Profiling of Anthers at the Mature Pollen Stage in Male-Sterile and Male-Fertile Potato Genotypes

Application of the GC-MS method led to an estimation of 193 compounds in the obtained profiles of potato anthers at the mature pollen stage. Further analysis of mass spectra and retention indexes with available libraries led to the identification of 70 metabolites. About the same amount (78 compounds) was determined up to the class level, most of which were sugars (Table S1.).

**Figure 1:**
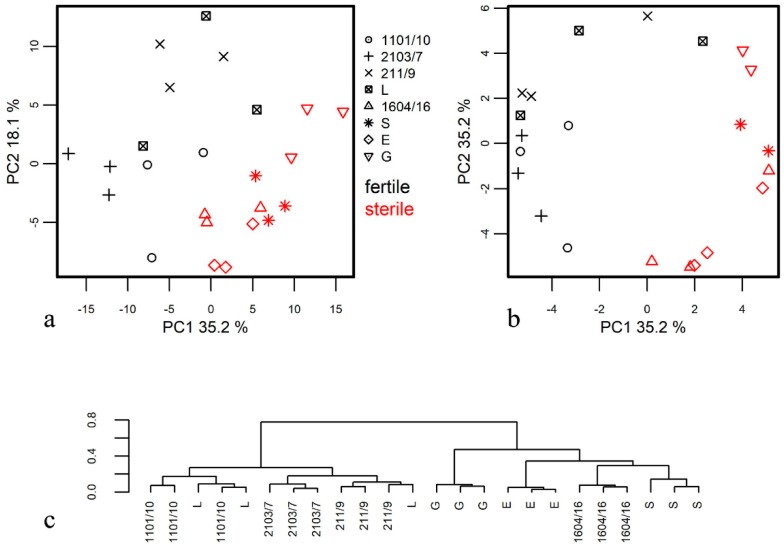
Clusterization of metabolite profiles of anthers at the mature pollen stage from male-fertile and male-sterile potato genotypes. (**a**) Principal Component Analysis (PCA) method of clusterization; (**b**) kernel PCA method of clusterization obtained with the hyperbolic tangent function; (**c)** dendrogram of hierarchical clustering obtained using the ratios of metabolite contents. L–cv. Lomonosovskij, S–cv. Sudarynja, E–cv. Evraziya. G–cv. Gusar, 1101/10, 1604/16, 2103/7, 211/9-breeding lines.

To find out differences in the metabolite profiles of male-sterile and male-fertile potato genotypes, several unsupervised methods of clusterization and dimension reduction were employed. The first approach of feature extraction was the PCA method (Figure 1a). Its application shows that samples are clearly clustered in the space of first two PCs according to their fertility/sterility characteristics. Statistical significance of differences was confirmed by Wilcox test for values of PC1 *p* = 1.02 × 10^−4^ and PC2 *p* = 0.008. Additionally, the hypothesis was tested by an analysis of variance using distance matrices with a permutation test (PERMANOVA) [42] where *p* ≤ 0.001.

At the same time, there is a certain tendency of clusterization according to genotype, though one much weaker and variable for tested genotypes. One of the important problems, which complicated data analysis, was nonlinearity. Application of the kernel analysis methods made available in the kernlab package (kPCA, [37]) is a way to overcome this problem. The most interesting result was obtained with the hyperbolic tangent function. In Figure 1b, it can be seen that observations are arranged in a circle, the first half of which is formed by metabolite profiles of anthers from male-fertile genotypes, and the second part by those of anthers from male-sterile genotypes (for PC1 values for fertile and sterile anthers differs *p* = 5.2 × 10^−6^). A significant difference between metabolic profiles of fertile and sterile genotypes is visualized in Figure S1a in the space obtained by the LLE (locally linear embedding) method (*k* = 8) (another way to decrease nonlinearity). In addition to analysis of the metabolite content as an initial parameter and their concentration as a dimension of the differences, it is possible to employ another value as a measure of proximity, such as the ratio of the content of metabolites and the ratios in concentrations. In case of logarithmic and standardized values, we assumed that concentration ratios as initial characteristics and Pearson correlation coefficients as distances would be equivalent. Figure S1b shows a graph of PCA scores with ratio analysis. This type of analysis clarifies visualization of differences between fertile and sterile genotypes. Further on, the application of the ratio of metabolite contents allows for the construction of a dendrogram with hierarchical clustering (Figure 1c). Application of correlation as distance gives a more precise clonal distinction. In addition, a similar picture emerges at Figure S1c, where profiles represented in the space were revealed with MDS (multidimensional scaling) from correlation distances.

Taken together, various methods of clusterization in anther metabolite profiles revealed significant differences resulting from fertility/sterility status, but not based on the sample pedigrees (Figure 1, Figure S1).

**Figure 2:**
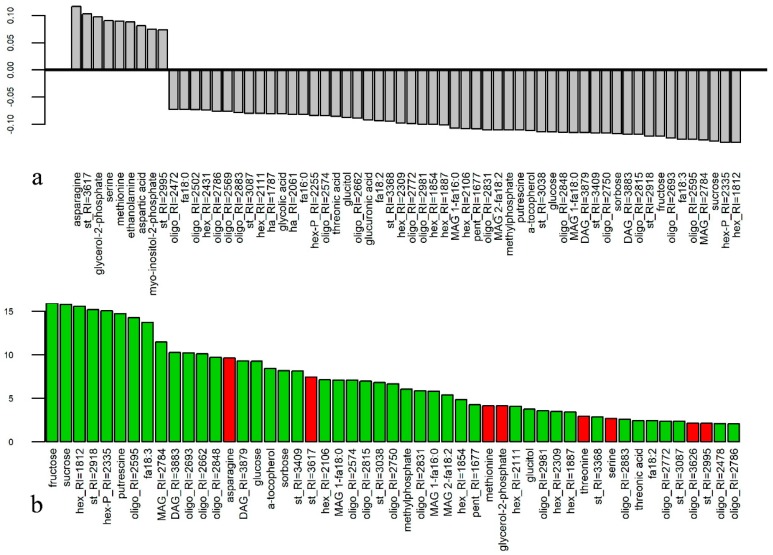
Features selection related to male sterility/fertility. (**a**) Loadings of a predictive component with VIP (variable importance in projection) values greater than 1. Positive values characterize metabolites with higher content in anthers of sterile genotypes; (**b**) Mean decrease accuracy (MDA) values obtained with the Random Forest (RF) method of classification. Green–higher in fertile, red–higher in sterile genotypes.

To search for systemic differences between the metabolite profiles between anthers from fertile and sterile genotypes, OPLS-DA (orthogonal partial least squares – discriminant analysis) and RandomForest (RF) methods were used. In the case of OPLS-DA, 24% of the variance is associated with the predictive component, R_2_X = 0.64, R_2_Y = 0.99, Q_2_Y = 0.93 Figure 2a presents the loadings of a predictive component with VIP (variable importance in projection) values greater than 1. Most metabolites have negative factor loads, which correspond to a greater content of metabolite in anthers of fertile genotypes. Carbohydrates, including glucose, fructose, and sucrose, rank among them. In addition, there are several fatty acids and some lipophilic compounds in this series. Not a single carbohydrate, despite their large number in the analyzed profiles, showed a positive factor of loading for VIP > 1. Positive factor loadings and, consequently, greater content of metabolite in anthers of sterile genotypes is characteristic of a number of amino acids: asparagine, aspartate, methionine, and serine. Employment of RF as the second method of classification showed that the OOB (out of bag) error was equal to 0, which gives an indication as to the reliability of the classification. Mean decrease accuracy (MDA) values (Figure 2b) have shown the leading role of sugars and acylglycerols in the diversity of metabolome profiles in anthers of fertile and sterile genotypes. Amino acids, whose level was higher in anthers with permanent tetrads, were of less importance. At the same time, RF for genotype differences had a high level of OOB error and, therefore, was not considered.

An additional RF classification was done with ratios of metabolite concentrations. Figure S2a shows the ratios with maximum MDA values. The detection of metabolites that made the greatest contribution to the differences between mature anthers of both fertile and sterile genotypes was provided by summing MDA ratios. Figure S2b presents a diagram of the largest sums. In general, the ratio of obtained dependencies was similar in both VIP and MDA, as revealed by an analysis based on metabolite concentrations. However, it can be noted that amino acids have been given higher importance.

**Figure 3:**
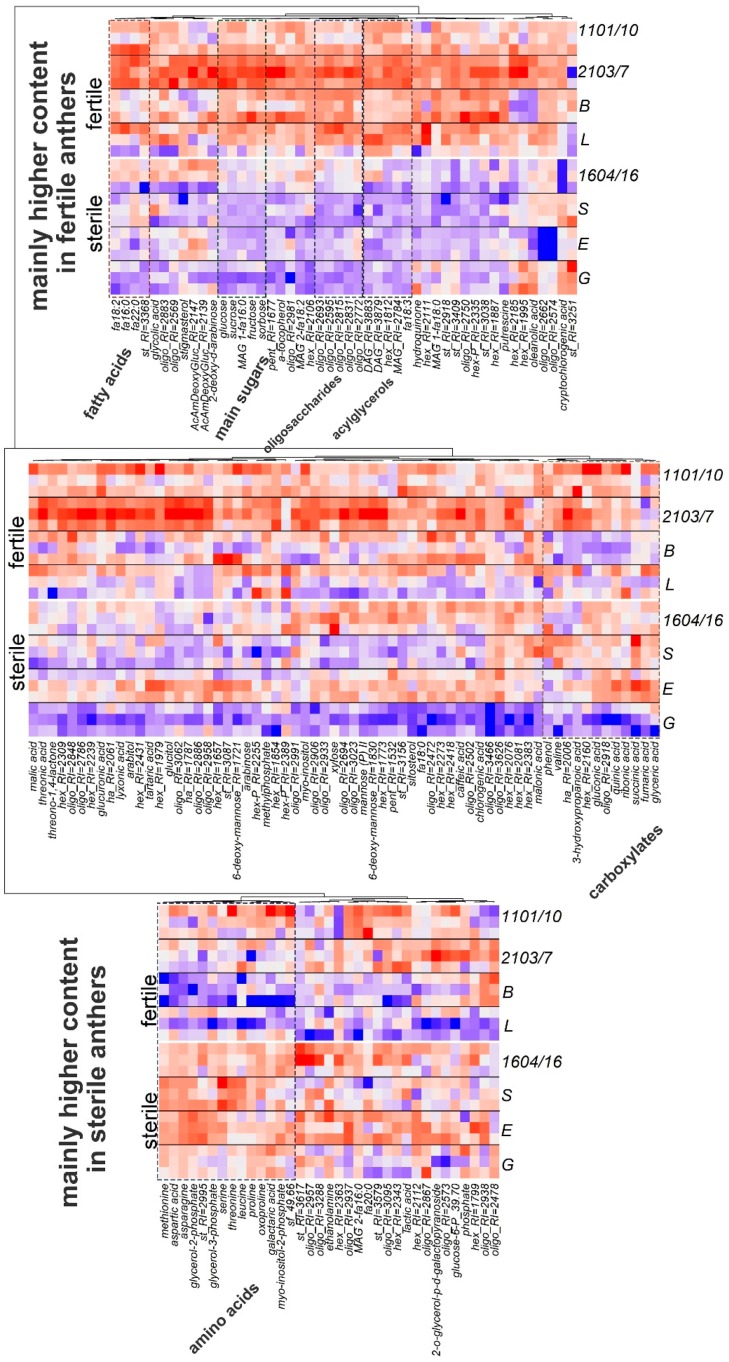
Heat map visualization of the content of identified metabolites in anthers at the mature pollen stage from fertile and sterile potato genotypes. This was combined with HCA (hierarchical cluster analysis) using correlation as distance (1-r) and the Ward method for cluster agglomeration. Data was standardized with red–higher content, blue–lower.

The content of the metabolites identified is visualized in the form of a heat map (Figure 3). In order to assess the effect of individual and clonal variability on the structure of interaction of metabolite pools, the heat map was combined with a dendrogram of hierarchical cluster analysis, where the correlation coefficient was used as a proximity factor.

The most significant alterations between profiles of male-fertile and male-sterile potato anthers related to such classes of metabolites as sugars, amino acids, and fatty acids. Fertility was characterized by enrichment with sugars (mono- and oligosaccharides) and lipophilic compounds (fatty acid and acylglycerols). Sterile anthers contained a higher amount of amino acids. Differences between genotypes inside fertile/sterile groups were not as strictly visualized.

**Figure 4:**
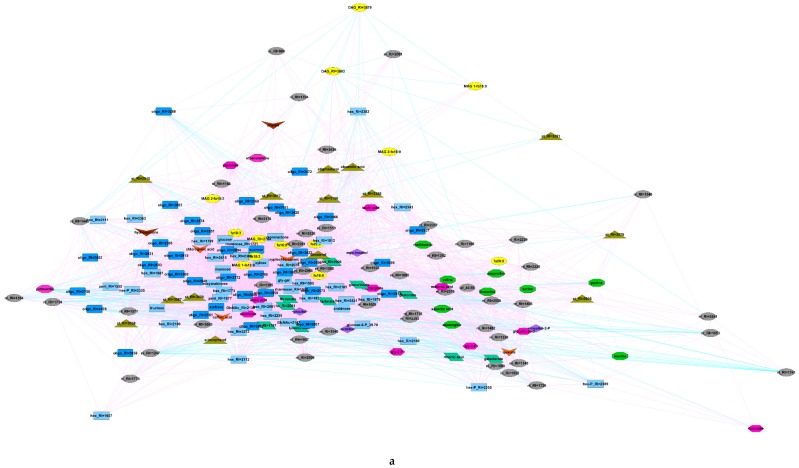

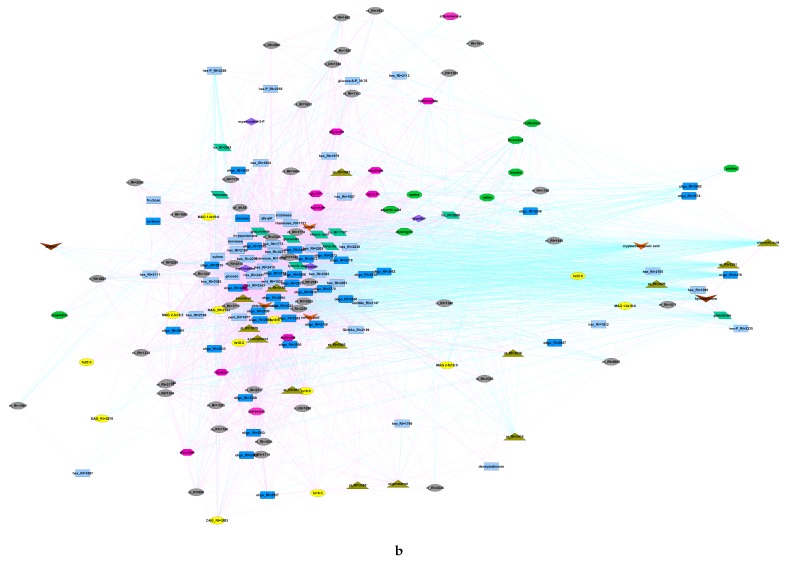
Metabolomic networks of anthers at the mature pollen stage of male-fertile (**a**) and male-sterile (**b**) genotypes. Yellow ovals–fatty acids and derivatives, blue rectangles–carbohydrates (bright–mono-, dark–oligosaccharides), green parallelograms–sugar acids, green octagons–amino acids, red hexagons–different small molecules, mainly carboxylates and other, olive triangles–sterols, brown concave quads–phenolic compounds, red concave quads–secondary metabolites, grey ovals–unidentified compounds.

Further analysis of relations between metabolites is represented as networks (Figure 4), where the nodes correspond to metabolites and the edges to correlation bonds. In this case, the stronger the connection, the shorter the edge. The network of fertile metabolites (Figure 4a) was characterized by the predominance of positive links. Sterility in the contrary intensified negative links (Figure 4b). Thus, structures of the nets vary distinctly between sterile and fertile genotypes, which indicated alteration in spectra of biochemical processes.

### 3.2. Assessment of the Frequency of Abnormalities in the First and the Second Meiotic Divisions

**Figure 5:**
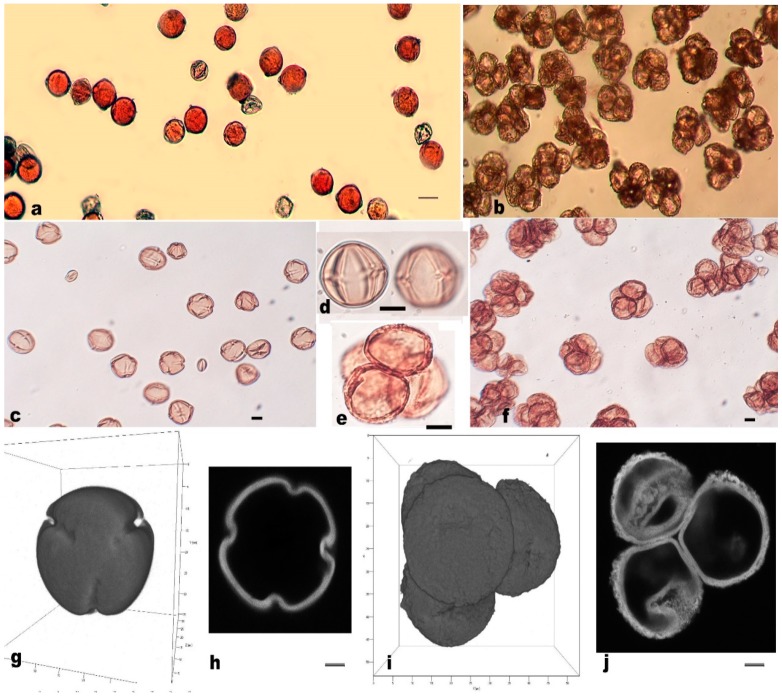
Light microscopy (LM) and confocal laser scanning microscopy (CLSM) images of male-fertile and male-sterile pollen grains. a, b–LM images of pollen, stained with acetocarmine from male-fertile cv. Lomonosovskij (**a**) and male-sterile line 1604/16 (**b**) potato genotypes. c-f–LM images of acetolysed pollen grains from male-fertile cv. Lomonosovskij (**c**,**d**) and male-sterile cv. Sudarynja (**e**,**f**). Scale bar–10 µm. g-j–CLSM images of acetolysed pollen grains from male-fertile cv. Lomonosovskij (**g**,**h**) and male-sterile line 1604/16 (**i**,**j**). Reconstructed pollen images (**g**,**i**) and optical sections through the pollen (**h**) and tetrads (**j**). Scale bar–5 µm.

Meiocytes from male-sterile and male-fertile genotypes have been compared at different developmental stages at the first and the second meiotic divisions. Most meiotic irregularities lead to the loss or gain of individual chromosomes both in male-sterile and fertile genotypes (Figure S3 and Table S2). A comparison of all stages of microsporogenesis in fertile and sterile potato genotypes demonstrates the absence of significant differences in the frequencies and the type of meiotic irregularities before the earlier tetrad stage. Thus, meiotic aberrations could not result in the formation of completely sterile pollen. In male-fertile genotypes, at the late tetrad stage, free microspores were released and they developed into functional pollen as was demonstrated in the pollen stainability test with acetocarmine (Table 1, Figure 5a). Whereas in male-sterile genotypes, tetrads failed to disintegrate in microspores and they remained integrated in ‘‘permanent’’ tetrads and lost their fertility (Figure 5b,e,f).

### 3.3. Measurement and Morphological Observation of Pollen Grains

For further morphological observation, pollen grains were taken from anthers and were acetolysed according to Erdtman [46]. In the three fertile genotypes (Lomonosovskij, 2103/7, 1101/10), four-colporate pollen grains predominated, and 98% of three-colporate pollen grains were found in the fertile 211/9 line (Table S3, Figure 5c,d,g,h). The comparison of the whole mass of data revealed that fertile pollen grains had a larger size than in sterile ones while exine thickness was the opposite–larger in sterile pollen grains gathered in ‘‘permanent’’ tetrads (Table S3). According to t-Student criteria, these differences were significant (*p* < 0.001).

The appearance of ‘‘permanent’’ tetrads in the male-sterile genotypes may indicate a violation of callose deposit. Detection of callose was provided by aniline blue staining widely used for this aim. It is characterized by fluorescence in a diapason of 495–510 nm. The obtained results did not reveal a difference between fertile and sterile pollen (Figure S4). To avoid the autofluorescence interfering, fertile and sterile pollen fluorescence spectrums were scanned for both stained and non-stained pollen. Spectrum maxima were detected at 475 nm (Figure S4).

After acetolysis, most microspores in the permanent tetrads remained fused together. The percent of monads varies from 0.9 in cv. Gusar up to 11.9 in the 1604/10 line. The fusion of pollen grains was prompted by the inner walls of the exine. Both LM and CLSM analysis revealed that the sterile potato genotypes showed a thickening of the sporoderm and anomalies in exine morphology. CLSM studies have shown that the exine thickness of male-fertile genotypes was approximately half that of male-sterile genotypes (Figure 5, Table S3). Aside from that, the main differences in sterile lines were the absence or degradation of apertures (Figure 5, Table S3).

## 4. Discussion

Any stage of plant development, including formation of reproductive organs, is accompanied by rearrangement in metabolic nets. The classic biochemical approach reveals the importance of carbohydrate balance during anther and pollen formation [48]. The decrease in sugar concentration leads to disturbances in reproduction and even sterility [49,50,51]. This might result from changes in gene expression, enzyme activity or intensity of assimilate transport [20,23,52]. Different expression patterns of gene families involved in carbohydrate metabolism have been identified between the anthers of male-sterile mutants and wild type plants for different plant species (rice [53] and cotton [54]). Any interruption in the sugar metabolism caused by stress factors led to pollen abortion [49]. Modulation of these genes expression is assumed to be a means of engineering sterility [21].

A recent untargeted metabolomics approach was employed to reveal alterations during anther development in tea plants and wheat [55,56] and differences between developing vegetative and reproductive organs in the potato [57]. Analysis of 146 metabolites throughout wheat anther development from tetrad to late uninucleate stage showed significant enrichment in numerous nutrient substances (including lipids, carbohydrates, and others). Special attention was paid to carbohydrates. Glucose and l-threose levels increased gradually from the tetrad stage to the binucleate and decreased slightly at the trinucleate stage, while maltose and fructose exhibited a steep and opposing tendency from the bi- to trinucleate stages. These data indicate that serious alterations took place in glycometabolism in anthers, a process assumed to result in starch synthesis.

Metabolome profiling allowed detection of changes in the balance of other compounds during anther development [56]: amino acids (about 65% of detected), organic acids (36 out of a total of 39), and fatty acids (palmitic, stearic, and linolenic). Similarly, the comparative analysis of metabolites profiles for the flower of the tea plant during development revealed alterations for 72 metabolites, including sugars, organic acids, and flavonoids [55]. Active metabolism in anthers was assumed from the comparison of metabolite profiling of the diploid cultivated potato *Solanum phureja* flowering plants [57]. Anthers at the mature pollen stage, in comparison to leaves, contain higher concentrations of fatty acids (16:0, 18:0, 18:2, 18:3), amino and organic acids (alanine, asparagine, tryptophan, tyrosine, valine, malate, malonate, oxalate, etc.), a number of secondary compounds (chlorogenic acid, quinic acid, cycloartenol, etc.), and sugars (sucrose, hexose, glucose, sugar phosphates, sugar alcohols, etc.). This indicates an active metabolism taking place in anthers even at late stages of development and diversity of anther metabolism from other potato plant organs.

GS-MS analysis was chosen in this study to examine the specific metabolic profile of mature anthers in male-sterile and male-fertile potato plants. About 200 (192) metabolites were uncovered (see Table S1). Sugars were the most representative group, a finding that correlates with earlier results. Among them were about 40 monosaccharides and their derivatives, 35 di- and tri-saccharides and their derivatives, a half dozen sugar acids and sugar alcohols. Additionally, a dozen amino acids, about a dozen fatty acids and acylglycerols, about half a dozen terpenes and many intermediates of various metabolic pathways were identified.

The further comparison of metabolite profiles of fertile and sterile potato genotypes revealed strict differences that were estimated with PCA analysis. Fertility/sterility status appeared to be more significant than pedigree relations even among inter-genotype distinctions (Figure 1, Figure S1). The effect was more pronounced if, instead of metabolite concentrations, metabolite ratios were analyzed. Most of the metabolites that showed a higher content in anthers of fertile genotypes were carbohydrates, including glucose, fructose, and sucrose. In addition, there were several fatty acids and other lipophilic compounds in this row. Positive factor loadings and, consequently, greater content in anthers of male-sterile genotypes is characteristic of a number of amino acids: asparagine, aspartate, serine, and methionine. At the same time, no amino acid showed a higher concentration in anthers of fertile genotypes (Figure 2 and Figure 3).

Alterations in metabolic correlation were visualized in developed metabolic networks (Figure 4). From the existence of two regions corresponding to sugars and amino acids, it could be concluded that the fertility/sterility status affected the cross-relationship of metabolites of these compound classes in different ways. Nevertheless, the predominance of positive correlations possibly shows an importance of maintaining a certain level of metabolite pool ratios for the maintenance of metabolic processes. An important feature of the anthers of male-sterile genotypes is a bigger number of negative links. In particular, a small cluster appears (Figure 4b) whose metabolite corresponds to others with negative correlations. This cluster mainly consists of lipophilic compounds and a number of carbohydrates. Apparently, the metabolic segment related to these metabolites is more greatly affected by the condition of sterility.

Thus, in agreement with the literature, the metabolic profiling of the present investigation clarifies the importance of carbohydrates for pollen fertility. Anthers of male-sterile plants differ by a lower level of sugars such as sucrose, glucose, fructose (Figure 2 and Figure 3), and higher concentrations of amino acids such as asparagine, aspartate, methionine, proline, serine, and threonine. The opposite regulation of sugars and amino acids concentration during pollen formation was also observed in other cases. For example, during the formation of a petunia’s pollen grain, the level of sugars decreased and level of amino acids increased [58].

Since the accumulation of sugars and amino acids may play an osmoprotective role [59], their opposite regulation may also be the result of compensational adaptation to desiccation. Similarly, under heat stress in the case of tomato anthers, an increase in the content of a number of amino acids has been observed simultaneously with a decrease in the content of glucose and fructose [60]. In addition, it was shown that impairing mitochondrial electron transport chain activity in lily pollen cells leads to an increase in the content of amino acids [61]. Thus, an increase in the level of amino acids against the background of a reduction in sucrose pools and products of fructose and glucose metabolism can be a universal indicator of metabolic disturbances during the process of pollen formation. This suggestion corresponds well to generated metabolomic networks of anthers at the mature pollen stage of male-fertile and male-sterile potato genotypes (Figure 4).

The data compiled show that transport of assimilated carbon directed to forming anthers as well as enzymes involved in regulating the balance of mono- and disaccharides is highly important for the synthesis of insoluble carbohydrate, which is in use during further pollen germination. Decrement of starch content resulted in the loss of pollen viability and resulted in male sterility. The accumulated sucrose, which serves an important energy source, may fulfill the role of an osmolyte in protecting pollen membranes and proteins during pollen dehydration or during exposure to stress conditions [62,63].

Disturbances in carbohydrate accumulation might affect other synthetic process, for example, the deposition of callose (β-1,3-glucan) around the microspores during meiosis, or callose dissolution at the end of the tetrad stage. Recently, genes encoding enzymes of callose metabolism and callose wall degradation (β-1,3-glucanase encoding genes) have been identified in different plant species, for example, in Arabidopsis [13,14] and the tomato [15]. Mutations in some of these genes cause the outer walls of the microspores to become fused at the end of meiosis, resulting in “arrested” microspores (“permanent tetrads”) and leading to complete male sterility. This study was directed toward a metabolomic analysis of potato anthers at the mature pollen stage, where callose should have already disappeared in fertile plants [64]. The absence of a fluorescence peak at 510 nm both in fertile and male-sterile potato genotypes was confirmed by CLSM analysis (Figure S4). The acetolysis method employed resulted in removing both internal contents of pollen grains, including intine, and the external possible organic shells envelope, including callose. However, it did not indicate degradation in ‘permanent tetrads’, which might also indicate the absence of callose between the fused microspores in male-sterile potato genotypes. It is known that the appearance of male sterility traits in the potato might be determined by nuclear-cytoplasmic interactions [10,11]. Biochemical changes, revealed in the present study, indicate the complexity of these interactions in the formation of tetrad sterility in the potato.

Based on results obtained in the present study, all male-sterile genotypes possessing the same W/gamma cytoplasm type were characterized by ‘permanent tetrads’, smaller pollen grain size in these tetrads, thicker exine, and the absence of apertures in pollen grains. All together, these changes indicate irregularities in exine formation. The pollen wall has a complex chemical composition, including callose, sporopollenin, polysaccharides, pigments, and others. The sporopollenin layer is resistant to strong reagents, acids, and alkalis and ensures protection of pollen grains from environmental influences. Numerous studies have shown that the sporopollenin mainly consists of a very long chain fatty acids, their polyhydroxylated derivatives and phenolic compounds, suggesting that the lipid metabolism is critical for sporopollenin biosynthesis and exine formation [25,27,65,66,67]. Thus, alterations in the balance of fatty acids and other lipophilic compounds might interfere with sporopollenin synthesis and affect pollen sterility. Our results demonstrate some changes for a group of fatty acids and other lipophilic compounds such as C18 and C16 fatty acids, acylglycerols, sterols, and others, which were less represented in sterile anthers (Figure 3). This phenomenon indicates deviations in sporoderm formation and abnormalities in sporopollenin deposition (Figure 5). It is suggested that the callose wall acts as a template for the formation of the pollen cell wall [64]. Recent investigations have shown various deviations in exine structure [68] and sporopollenin biosynthesis is now being actively examined; thus, notable progress has been made in understanding exine formation [27].

## 5. Conclusion and Out Look

Overall, the data of metabolite profiling estimated differences in the metabolic nets of male-fertile and male-sterile potato genotypes with W/gamma cytoplasm type. Sterility is characterized by a significant decrease in the carbohydrate pool and an increase in amino acid content in anthers at the stage of mature pollen. The number of alterations was found in a pool of fatty acids and lipophilic compounds. This phenomenon is accompanied by a change in the morphology of cell walls. Its origin might be due to the synthesis and deposition of callose and likely depends on the quality of sporopollenin. However, this assumption requires additional study devoted to the earlier stages of microsporogenesis independent from the specification of polymer synthesis. The biochemical alterations observed in the present study are under nuclear control and thus shed light on the “genetic-cytoplasmic” (or nuclear-cytoplasmic) male sterility postulated for potato.

## Figures and Tables

**Table 1:** Plant material used in the study.

#	Genotype	Designations	Pedigree Information *	Cytoplasm Type *	% of Normal Pollen Grains Stained with Acetocarmine
**Male-sterile genotypes:**					
1	cv. Gusar	G	cv. Arosa × cv. Vdokhnoveniye	W/Gamma	0
2	cv. Sudarynja	S	8889/3 × 89181/6	W/Gamma	0
3	cv. Evraziya	E	95100/27 × 943/9	W/Gamma	2.3%
4	1604/16	1604/16	95100/27 × 943/6	W/Gamma	0
**Male-fertile genotypes:**					
5	1101/10	1101/10	cv. Charodej × 943/9	W/alpha(D)	76.2%
6	211/9	211/9	cv. Charodej × 943/6	W/alpha(D)	90.9%
7	2103/7	2103/7	-	W/alpha(D)	89.9%
8	cv. Lomonosovskij	L	89287/1 × 8334/20	W/alpha(D)	73.0%

* Pedigree records and cytoplasm types (which were determined according to [4]) were published recently [28]. Pollen viability (in the present study) has been estimated in the anthers isolated from the plants used in metabolome analysis.

## Supplementary Materials

The following are available online at http://www.mdpi.com/2218-1989/9/2/24/s1, Figure S1: Clusterization of metabolite profiles of anthers at the mature pollen stage from male-fertile and male-sterile potato genotypes, Figure S2: RF classification with ratios of metabolites concentrations, Figure S3: Meiosis in PMC of *S. tuberosum* – male-fertile (cv. Lomonosovskij) and male-sterile genotypes (cv. Sudarynja, cv. Gusar), Figure S4: Typical fluorescence spectra obtained from non-stained (a) and stained (b) with aniline blue pollen, Table S1: Arbitrary concentrations of metabolites in anthers of sterile and fertile varieties of *S. tuberosum*, Table S2: Characteristics of meiosis in *S. tuberosum* male-sterile and -fertile varieties, Table S3: Morphological parameters of pollen grains in genotypes differed in its fertility/sterility status.
