# Supplementary material for: Metabolic Alterations in Male-Sterile Potato as Compared to Male-Fertile
Source: Metabolites. 2019 Feb 1;9(2):24. doi: 10.3390/metabo9020024 (PMC6409681; doi:10.3390/metabo9020024)
Supplement: Supplementary file 1 [file metabolites-09-00024-s001.zip › Supplementary material/Supplementary figure legends.pdf]

Supplementary figure legends

Fig. S1. Clusterization of metabolite profiles of anthers at the mature pollen stage from male fertile and male sterile potato genotypes. a) PCA loadings; b) LLE method (k = 8); c) PCA scores with ratio analysis; d) profiles represented in the space revealed with MDS.

Fig. S2. RF classification with ratios of metabolites concentrations. a) maximum MDA values; b) MDA ratios summing up.

Fig. S3. Meiosis in PMC of *S. tuberosum* – male fertile (cv. Lomonosovskij) and male sterile genotypes (cv. Sudarynja, cv. Gusar)

Fig. S4. Typical fluorescence spectra obtained from non-stained (a) and stained (b) with aneline blue pollen.
