# Supplementary material for: Metabolic Alterations in Male-Sterile Potato as Compared to Male-Fertile
Source: Metabolites. 2019 Feb 1;9(2):24. doi: 10.3390/metabo9020024 (PMC6409681; doi:10.3390/metabo9020024)
Supplement: Supplementary file 1 [file metabolites-09-00024-s001.zip › Supplementary material/Supplementary Figure S3.pdf]

Figure S3.

cv. Lomonosovskij	cv. Sudarynja	cv. Gusar
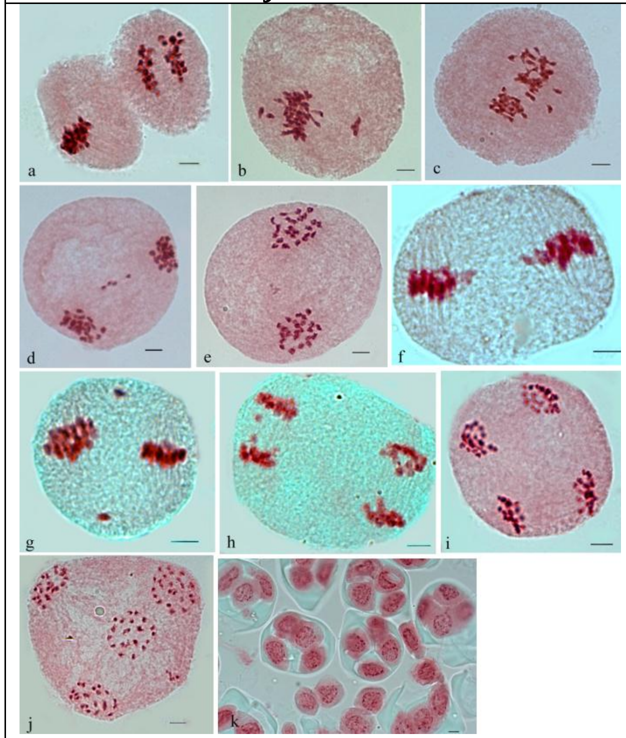	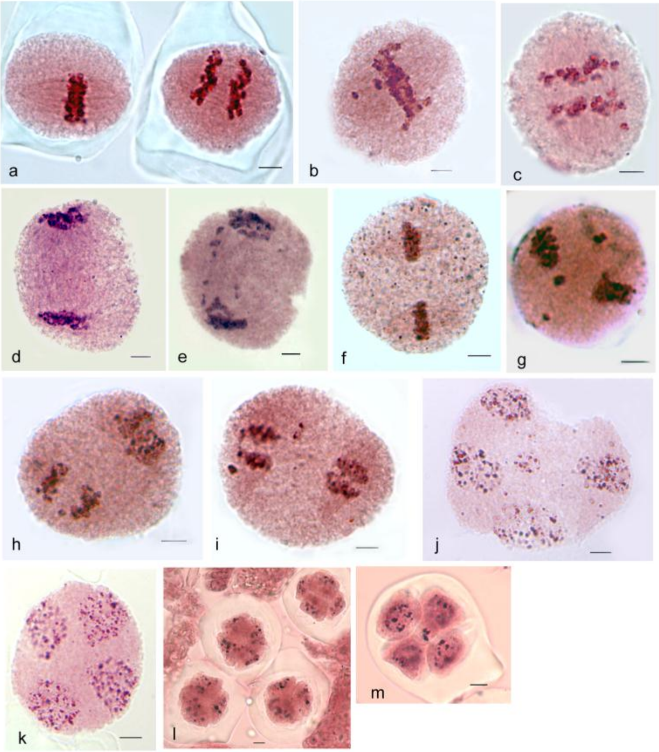	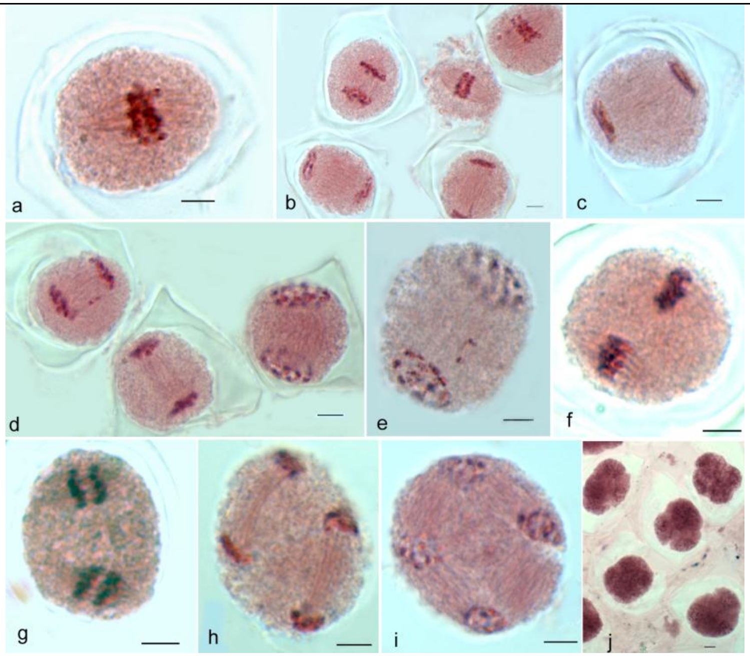
PMCs with (a) regular M I and AI;<br>(b) 4 non oriented chromosomes at M1;<br>(c) 2 lagging chromosomes at MI;<br>(d) lagging chromosomes at TI;<br>(e) normal TI;<br>(f) regular MII;<br>(g) 2 non oriented chromosomes at MII;<br>(h) 2 lagging chromosomes at AII;<br>(i) regular TII;<br>(j)) micronucleus formation at TII;<br>(k) tetrads of microspores.	PMCs with (a) regular M I and AI;<br>(b) PMC with 1 non oriented chromosome at M1;<br>(c) 1 precocious chromosome at AI;<br>(d) the normal T1; (e) lagging chromosomes at TI;<br>(f) regular MII;<br>(g) non oriented chromosomes at MII;<br>(h) lagging chromosome at AII;<br>(i) 2 non oriented chromosomes at AII;<br>(j) micronucleus formation at TII; (k) normal TII;<br>(l) tetrads of microspores;<br>(m) tetrad with 1additional microspores derived from micronuclei.	(a) PMC with regular M I;<br>(b) the group of PMCs with regular MI, AI, TI and abnormal MI with 1 non oriented chromosome;<br>(c) regular TI;<br>(d) PMCs with regular TI and TI with 1 lagging chromosome;<br>(e) TI with 1 non oriented chromosome;<br>(f) regular MII;<br>(g) regular AII;<br>(h-i) regular TII;<br>(j) tetrads of microspores.
