# Supplementary material for: Metabolic Alterations in Male-Sterile Potato as Compared to Male-Fertile
Source: Metabolites. 2019 Feb 1;9(2):24. doi: 10.3390/metabo9020024 (PMC6409681; doi:10.3390/metabo9020024)
Supplement: Supplementary file 1 [file metabolites-09-00024-s001.zip › Supplementary material/Supplementary Table S2.pdf]

Table S2. Characteristics of meiosis in *S. tuberosum* male sterile and fertile varieties

Variety	Phase of meiosis	No of analyzed PMC	Number of normal PMC /%	Number of abnormal PMC / %	Type of abnormalities
Lomonosovskij male fertile	M I	186	105/56,4	81/43,6	1-5 non oriented chromosomes precocious chromosomes (1-2 pares)
	A I	82	44/53,7	38/46,3	precocious, lagging chromosomes (1-2 pares), 1-4 non oriented chromosomes
	T I	130	87/66,9	43/33,1	1-4 non oriented chromosomes
	M II	60	43/71,7	17/29,3	precocious, lagging chromosomes (1-2); 1-4 non oriented chromosomes
	A II	29	21/72,4	8/27,6	2-4 non oriented chromosomes
	T II	92	75/81,5	17/18,5	1-4 micronuclei
	Tetrads	120	98/81,7	22/18,3	Tetrads with 1-2 additional microspores derived from micronuclei
Sudarynja male sterile	M I	358	198/55,3	160/44,7	1-6 non oriented chromosomes; precocious chromosomes (1-2 pares)
	A I	86	47/54,7	39/45,3	precocious, lagging chromosomes; non oriented chromosomes (1-4);
	T I	40	34/85,0	6/15,0	1-4 non oriented chromosomes lagging chromosomes(1-4)
	M II	37	20/54,1	17/45,9	2-4 non oriented chromosomes
	A II	18	11/61,1	7/38,9	2-4 non oriented chromosomes lagging chromosomes
	T II	93	58/62,4	35/37,6	1-4 micronuclei
	Tetrads	163	138/84,7	25/15,3	Tetrads with 1-2 additional microspores derived from micronuclei
Gusar male sterile	M I	598	538/90,0	60/10,0	1-3 non oriented chromosomes precocious chromosomes
	A I	329	306/93,0	23/7,0	1-3 lagging chromosomes; non oriented chromosome (1)
	T I	272	262/96,3	10/3,7	non oriented chromosome (1), lagging chromosomes(1-4)
	M II	144	140/97,2	4/2,8	1-2 non oriented chromosomes
	A II	51	47/92,2	4/7,8	1-3 lagging chromosomes
	T II	34	33/97,1	1/2,9	1 micrunucleus
	Tetrads	88	88/100,0	0/0,0	

PMC – Pollen Mother Cells
