# Supplementary material for: Metabolic Alterations in Male-Sterile Potato as Compared to Male-Fertile
Source: Metabolites. 2019 Feb 1;9(2):24. doi: 10.3390/metabo9020024 (PMC6409681; doi:10.3390/metabo9020024)
Supplement: Supplementary file 1 [file metabolites-09-00024-s001.zip › Supplementary material/Supplementary Table S3.pdf]

Table S3. Morphological parameters of pollen grains in genotypes differed in its fertility/sterility status

| | Pollen grain size, µm | Tetrads after acetolysis, % | Aperture number | Exine thickness, µm |
|---|---|---|---|---|
| Genotype | Average value ± SD | | | Average value ± SD |
| Male fertile genotypes | | | | |
| 1101/3 | 25,20±2.46 | | 3 (2,4) | 1,40±0.45 |
| Lomonosovskij | 26,28±1.47 | | 4 (3,2) | 1,45±0.33 |
| 211/9 | 26,64±2.62 | | 4 (3) | 1,34±0.26 |
| 2103/7 | 26,47±2.34 | | 4 | 1,37±0.34 |
| Total fertile genotypes | 26.04 ± 0.24 | | | 1.39 ± 0.02 |
| | | | | |
| Male sterile genotypes | | | | |
| 1604/16 | 22,92±1.71 | 11.9 | not detected | 2,18±0.54 |
| Evraziya | 22,74±2.18 | - | not detected | 1,86±0.52 |
| Sudarynja | 24,90±2.74 | 6.3 | not detected | 2,99±0.89 |
| Gusar | 22,98±2.50 | 0.9 | not detected | 2,57±0.75 |
| Total sterile genotypes | 23.60 ± 0.23* | | not detected | 2.45 ± 0.06* |
| | | | | |

*Differences between pollen grain size and exine thickness of fertile and sterile genotypes are significant ($p < 0.001$).
