# Supplementary figures and images for: Metabolic Alterations in Male-Sterile Potato as Compared to Male-Fertile
Source: Metabolites. 2019 Feb 1;9(2):24. doi: 10.3390/metabo9020024 (PMC6409681; doi:10.3390/metabo9020024)

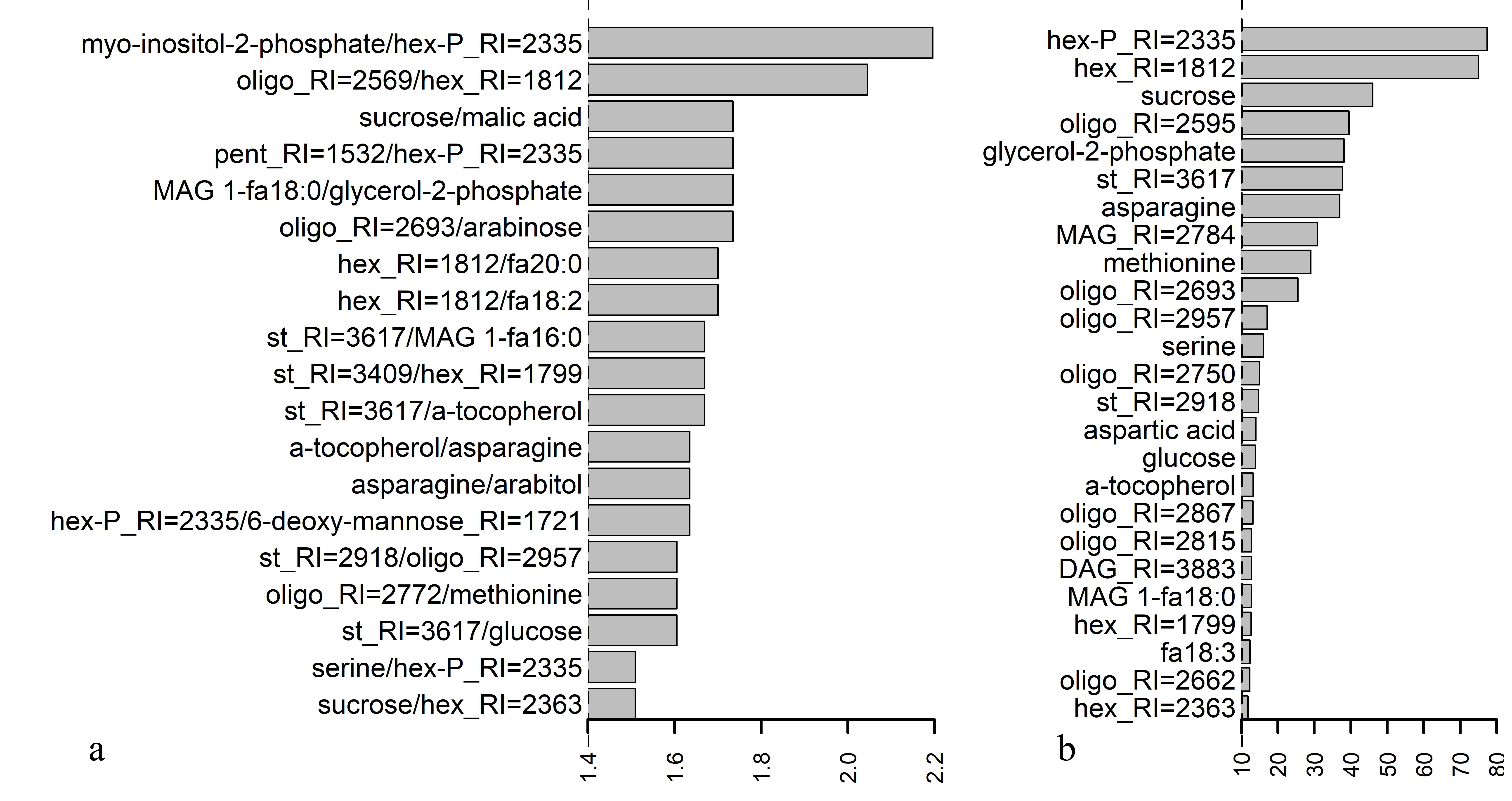

Supplement: Supplementary file 1 [file metabolites-09-00024-s001.zip › Supplementary material/Supplementary Figure S2.tif]

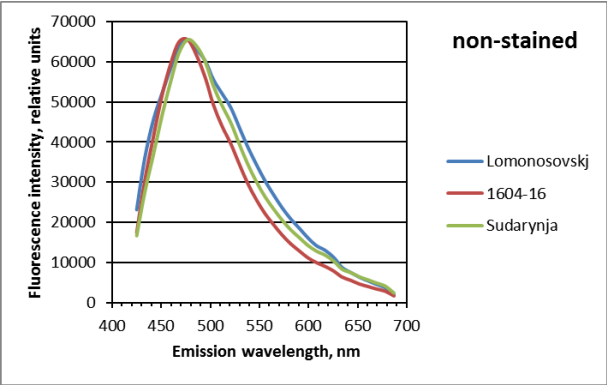

a

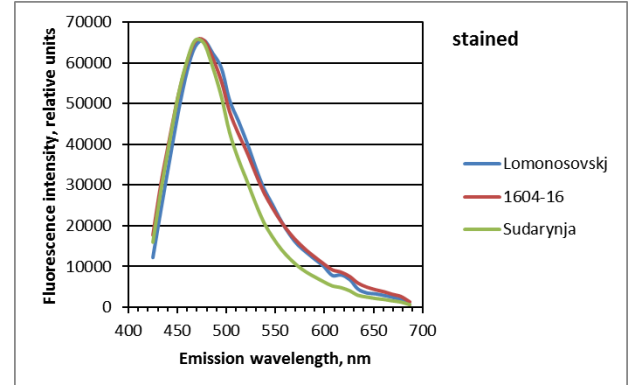

b

Supplement: Supplementary file 1 [file metabolites-09-00024-s001.zip › Supplementary material/Supplementary Figure S4.pdf]
